# Synthetic applications of the Cannizzaro reaction

**DOI:** 10.3762/bjoc.20.120

**Published:** 2024-06-19

**Authors:** Bhaskar Chatterjee, Dhananjoy Mondal, Smritilekha Bera

**Affiliations:** 1 Department of Chemistry, Nabadwip Vidyasagar College, West Bengal, India; 2 School of Chemical Sciences, Central University of Gujarat, Gandhinagar-382030, India

**Keywords:** Cannizzaro reaction, crossed-Cannizzaro, desymmetrization, Lewis acid catalyst, natural products

## Abstract

The Cannizzaro reaction has emerged as a versatile synthetic tool for the construction of functionalized molecules. Dating back to the 19th century, this reaction, though initially used for the synthesis of an alcohol and acid functionality from aldehydes, has henceforth proven useful to generate diverse molecular entities using both intermolecular and intramolecular synthetic strategies. Immense applications in the synthesis of hydroxy acids and esters, heterocycles, fused carbocycles, natural products, and others with broad substrate scope have raised profound interest from methodological and synthetic standpoints. The ongoing development of reagents, solvents, and technologies for the Cannizzaro reaction reflects the broader trend in organic synthesis towards more sustainable and efficient practices. The focus of this review is to highlight some recent advances in synthetic strategies and applications of the Cannizzaro reaction towards the synthesis of potentially useful molecules.

## Introduction

**Figure 1:**
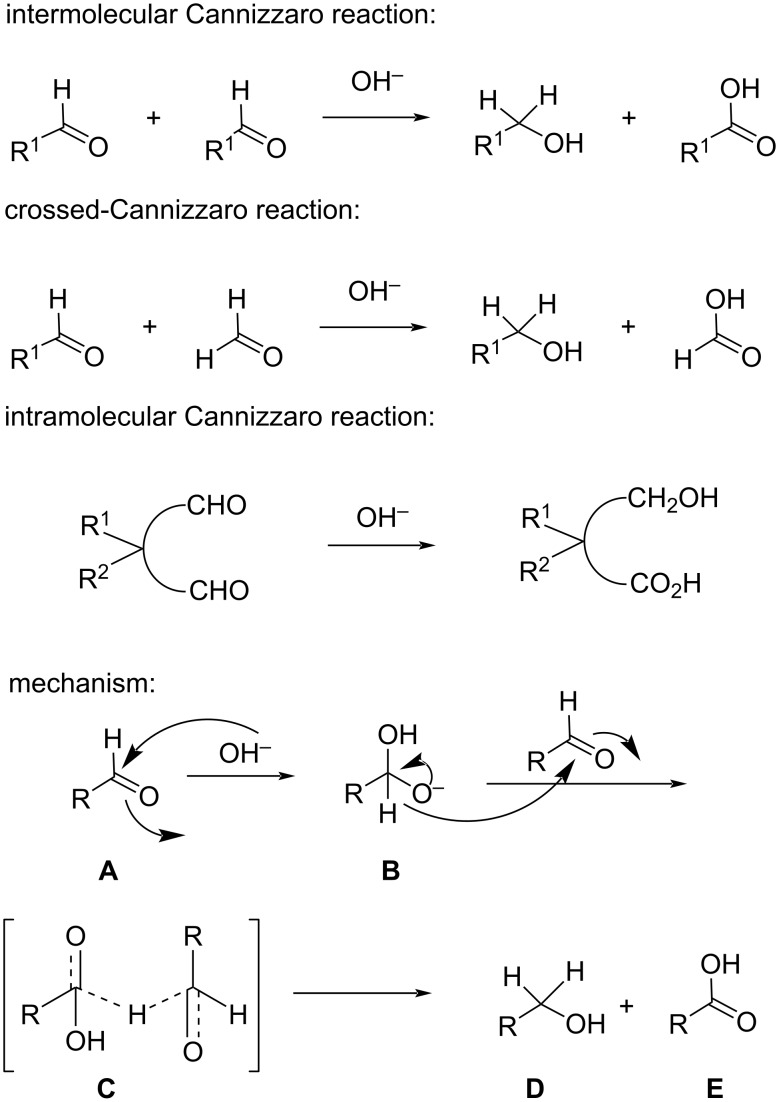
Types and mechanism of the Cannizzaro reaction.

The synthesis of functionalized molecules with structural complexity has always been a challenge to synthetic chemists. The Cannizzaro reaction, in its simplified form, focuses on the base-induced disproportionation of two molecules of a non-enolizable aromatic and/or aliphatic aldehyde (without an α-hydrogen atom). These aldehydes undergo in the presence of concentrated alkali or other strong bases, a simultaneous oxidation and reduction sequence of two aldehyde molecules, forming an alcohol and an acid [1–4]. Since its discovery in 1853, the Cannizzaro reaction has emerged as an important reaction in synthetic organic chemistry with intermolecular, crossed, and intramolecular versions as demonstrated by numerous applications. Notably, the Cannizzaro reaction has come across with subtle developments and changes in base modifications leading to compounds of potential interest [5–6]. The intermolecular Cannizzaro reaction is a chemical process in which two molecules of a non-enolizable aldehyde (2R^1^CHO) are disproportionated by a base to produce a carboxylic acid (R^1^CO_2_H) and a primary alcohol (R^1^CH_2_OH). When a mixture of formaldehyde (HCHO) and a non-enolizable aldehyde (R^1^CHO) is treated with a strong base, the latter is preferentially reduced to the alcohol (R^1^CH_2_OH) while formaldehyde is oxidized to formic acid (HCO_2_H). Herein excess formaldehyde is used as a reductant. This variant is known as the crossed-Cannizzaro reaction. On the other hand, an intramolecular Cannizzaro reaction occurs when both aldehyde groups are present in a single molecule. In this scenario, one aldehyde group is reduced to the corresponding alcohol, while the other is oxidized to a carboxylic acid. The mechanistic pathway of the intramolecular, intermolecular, and crossed-Cannizzaro reactions is well-known and is depicted in Figure 1 [7–11]. As per contemporary mechanistic understanding of this disproportionation reaction, it involves the transfer of a hydride ion from a tetracoordinated intermediate (**B**), which is formed upon hydroxide addition to the aldehyde (**A**). The primary pathway of the reaction entails the rate-determining step of hydride ion transfer via either a linear or bent transition state (**C**) to a second molecule of aldehyde furnishing the corresponding alcohol (**D**) and acid molecule (**E)**.

## Review

### Modernization of the Cannizzaro reaction

Researchers have always aimed to improve the efficiency, selectivity, and sustainability of reaction processes. The development of reagents, solvents, and application of modern technology frame the Cannizzaro reaction as an important synthetic tool in organic synthesis.

Certain developments in the Cannizzaro reaction with regard to reagents, solvents, and technologies are worth mentioning. Generally, the classical version of the Cannizzaro reaction is conducted at elevated temperatures using stoichiometric amounts of alkali metal hydroxides or other strong bases, commonly NaOH, KOH, Ba(OH)_2_·8H_2_O or sodium ethoxide, etc. [12–18]. Such harsh conditions and the competitive formation of undesired side products have been the major limiting factors for the Cannizzaro reaction in the past several decades [19–22].

The base-mediated solvent-free Cannizzaro reaction was achieved through various methods, such as by grinding reactants in mechanochemical approaches and/or solid-supported bases. Minimizing the use of hazardous solvents not only reduces environmental impact but also simplifies product isolation and purification and improves the overall sustainability of the process [23–24]. However; specific reaction requirements ensure efficient and selective utilization of solvents.

Marvi and Talakoubi carried out the Cannizzaro reaction [25] using montmorillonite K-10 and KSF clays as recyclable and heterogeneous catalysts to catalyze the Cannizzaro reaction by 1,4-diazabicyclo[2.2.2]octane under microwave irradiation and solvent-free conditions giving the products in excellent yields within seconds. The solid clay applied in the first cycle can be recovered and reused in subsequent reactions. Reddy and coworkers carried out the Cannizzaro reaction of aromatic aldehydes to the corresponding alcohols in high yields by crossed-Cannizzaro reactions employing solid-supported KF-Al_2_O_3_ as catalyst [26] under microwave irradiation using solvent-free conditions.

The use of different phase-transfer reagents and catalysts such as benzyltriethylammonium chloride, tetrabutylammonium bromide and many others have had profound impacts on the Cannizzaro reaction. Entezari and Shameli [27] studied the effect of an ultrasonic wave on the Cannizzaro reaction catalyzed by a phase-transfer catalyst in the presence of KOH as the base. Canipelle et al. [28] put forward an improved Cannizzaro disproportionation of 4-biphenylcarboxaldehyde into the corresponding alcohol and carboxylic acid products employing cyclodextrins as the phase-transfer agent. A Cannizzaro desymmetrization reaction of tetraethylene glycol (TEG) was accomplished by Vida et al. [29] using a barium compound.

Different types of solvents were also applied for the Cannizzaro reactions. The use of aqueous or solvent-free conditions has played pivotal roles in terms of environmental consciousness and a greener reaction [30–31]. Daemi et al. employed polyurethane nanomicelles as an eco-friendly and efficient polymeric ionic solvent [32]. Morooka et al. used supercritical water for the catalys-free Cannizzaro-type reaction of acetaldehyde [33]. Thus, the originally developed Cannizzaro reaction was subjected to numerous modified techniques, which established the greener side of the reaction. The use of Lewis acid catalysis in this regard [34–39] played a significant role, which also suppressed the epimerization in the case of chiral molecules. Among the various Lewis acid catalysts such as ytterbium triflate, ZrO_2_, Cu(OT)_2_, MgBr_2_, LiBr, AlCl_3_, Fe and Ru catalysts have gained attention [40–43].

The Cannizzaro reaction has also found extensive use in the synthesis of bioactive and drug molecules [44–46]. Moreover, enzymatic transformations have been also observed for the synthesis of α-hydroxycarboxylic acids [47]. They are also used in the development of different nanoparticle preparations and other reactions for synthesis of bioactive compounds [48–50].

Green synthesis methodologies, such as microwave-assisted [51–53] and ultrasound-assisted reactions [54] are established techniques in green chemistry due to their potential benefits in terms of reduced reaction times, increased yields, and the ability to perform reactions under milder conditions than traditional methods. The Cannizzaro reaction, being a useful synthetic tool, has also been explored in this regard. The Cannizzaro reaction also finds extensive industrial use in synthesizing pentaerythritol, a crucial intermediate in manufacturing of alkyd resins and plasticizers [55]. The crossed-Cannizzaro reaction contributes to polyol production for polyester resin synthesis. In the field of fragrance and flavoring agents, it plays a vital role for the development of unique sensory compounds [56–57].

**Figure 2:**
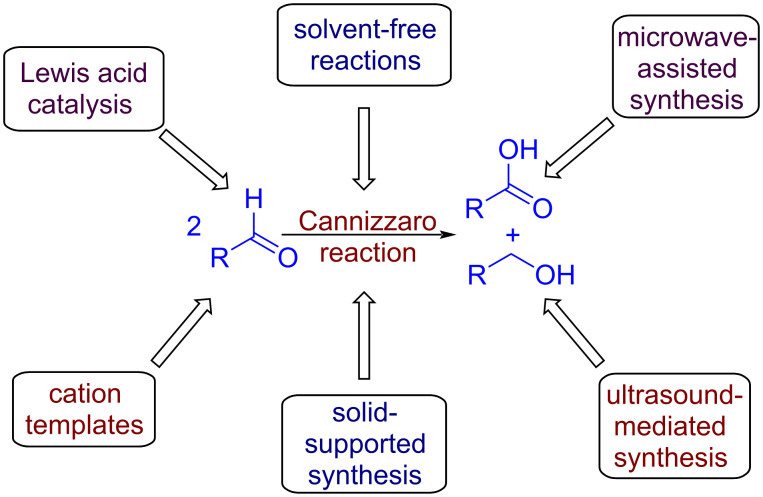
Various approaches of the Cannizzaro reaction.

The Cannizzaro disproportionation has also been observed in several electrochemical transformations [58] and during the electrocatalytic reduction of carbon dioxide [59]. A recent study by Liu et al. witnessed a competing Cannizzaro reaction during the electrochemical oxidation of furfural [60]. On the other hand, 5-hydroxymethyl-2-furancarboxylic acid (HMFCA) and dihydroxymethylfuran (DHMF), obtained via the Cannizzaro disproportionation of 5-(hydroxymethyl)furfural, were electrochemically oxidized to 2,5-furandicarboxylic acid (FDCA), a monomer used for biopolymer production [61]. Other modifications include the use of ortho-substituted aromatic amines as a base in the Cannizzaro reaction and others (Figure 2) [62].

**Figure 3:**
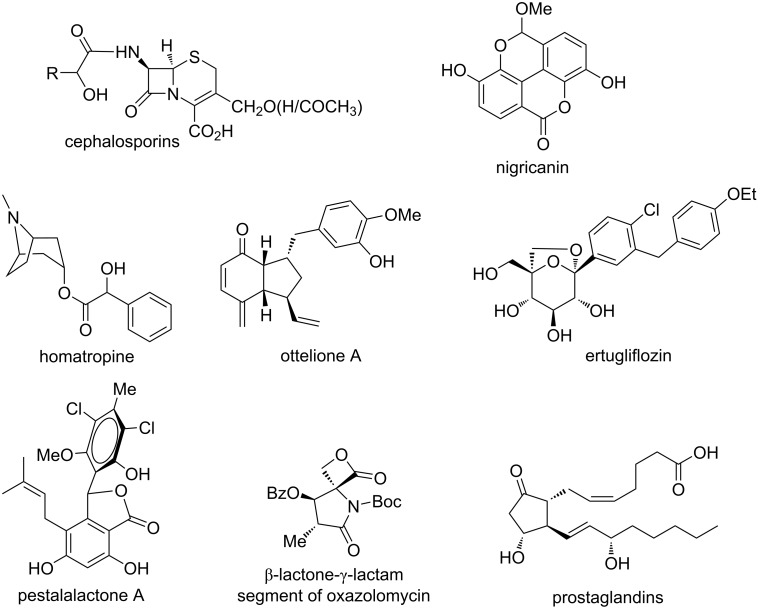
Representative molecules synthesized via the Cannizzaro reaction.

The synthesis of several useful molecular entities [63–64] and synthons of biologically relevant compounds, which include prostaglandins [65], β-lactams [66–67], homatropine [68–69] among many more [70] (Figure 3), reinforce the importance of the Cannizzaro reaction. Modifications of both intermolecular and intramolecular Cannizzaro reactions have been observed in numerous methodologies, such as Lewis acid catalysis, desymmetrization of symmetrical dialdehydes, synthesis of natural products, and building blocks. These modifications constitute the main highlight of this review. The use of modern technology and newer strategies aiming towards industrial benefit is the goal for the future [71–72]. Herein, we discuss recent advances in the Cannizzaro reaction, focusing on the synthetic developments of natural products and important building blocks in the last two decades.

### Applications of the Cannizzaro reaction in organic synthesis

The Cannizzaro reaction has been significantly important in synthetic organic chemistry due to its ability to provide a convenient route for the synthesis of alcohols and carboxylic acids from aldehydes. This disproportionation reaction has evoked numerous developments and applications. The Cannizzaro reaction proved to be particularly valuable in cases where other methods of oxidation or reduction might be challenging or impractical. The present discussion focuses on some recent synthetic advances and their application in biologically active compounds.

#### Lewis acid-catalyzed intramolecular Cannizzaro reaction

**Scheme 1:**
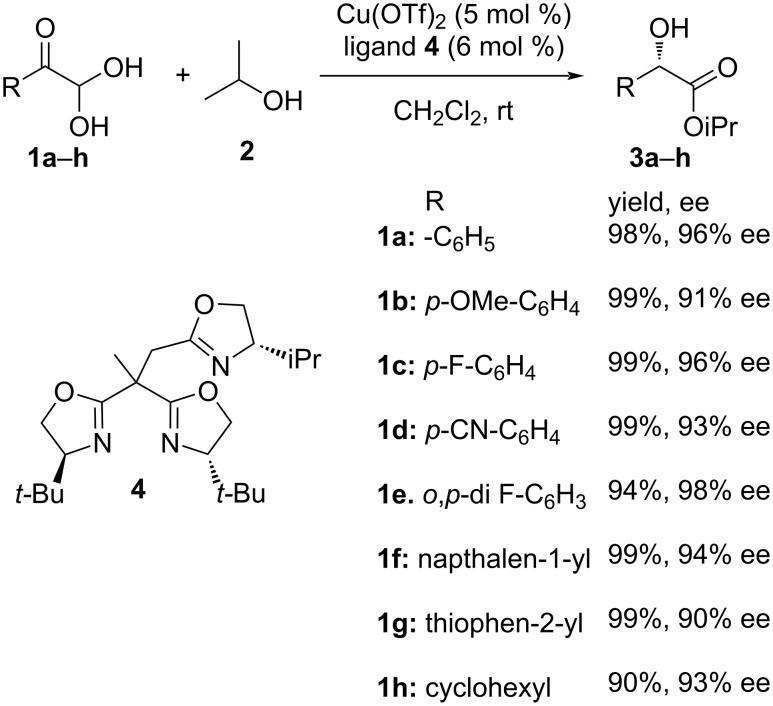
Intramolecular Cannizzaro reaction of aryl glyoxal hydrates using TOX catalysts.

Wang et al. [73] depicted a highly enantioselective intramolecular Cannizzaro reaction of aryl and alkyl glyoxals **1a**–**h** and alcohol **2** using trisoxazoline (TOX) ligand (**4**)/copper catalysts to furnish the requisite mandelic esters **3a**–**h** in good yields (greater than 90%) and high enantioselectivity. This was observed in the wide substrate scope as represented in the table below. The yields and selectivity were found to be superior compared to bisoxazoline (BOX) ligands, which was attributed to the steric bulk imparted by the ligand at the stereoinduction step. Increasing the steric size of the alcohol also contributed to the increased enantioselectivity of the resultant product (Scheme 1).

**Scheme 2:**
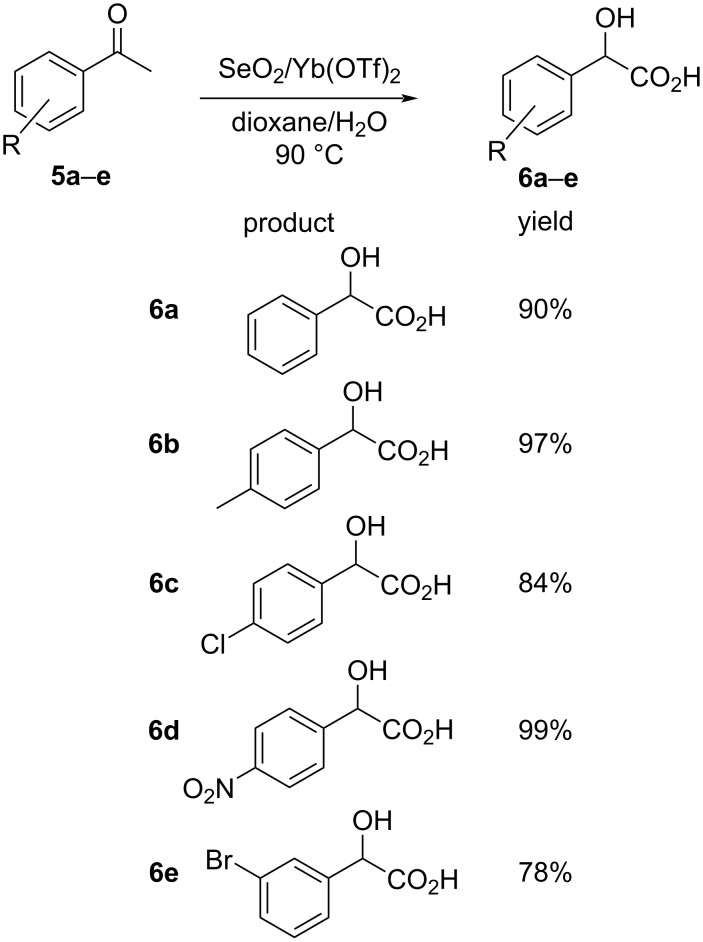
Intramolecular Cannizzaro reaction of aryl methyl ketones using ytterbium triflate/selenium dioxide.

A one-pot oxidation–Cannizzaro reaction of aryl methyl ketones to mandelic acid derivatives was observed in the presences of ytterbium triflate as the catalyst. The intramolecular reaction sequence employed a SeO_2_/Yb(OTf)_3_ combination to affect the in-situ oxidation of the aryl methyl ketones **5** to the corresponding aryl glyoxal with concomitant rearrangement of the aryl glyoxal to the target α-hydroxycarboxylic acid derivatives **6**, catalyzed by Yb(OTf)_3_. The simple process reflects the generality of the methodology with yields ranging from 78–99% as represented below (Scheme 2) [34].

**Scheme 3:**
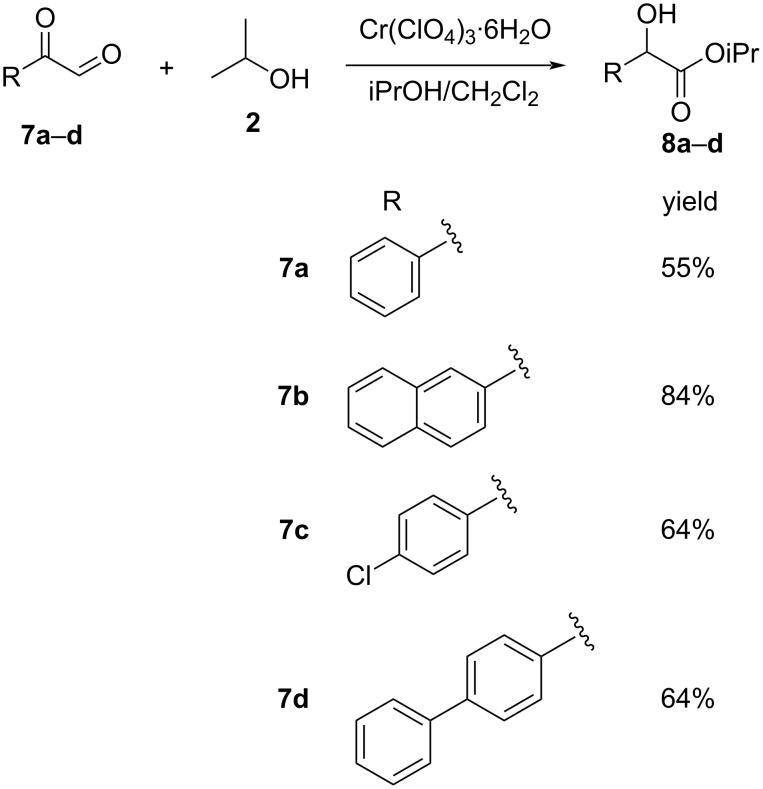
Intramolecular Cannizzaro reaction of aryl glyoxals using Cr(ClO_4_)_3_ as catalyst.

Morken and coworkers [36] set forth an intramolecular Lewis acid-mediated Cannizzaro reaction of aryl glyoxals **7** at room temperature using appropriate chromium or copper catalysts. The strategy afforded moderate to good yields of Mandelic esters **8** in the presence of Cr(ClO_4_)_3_ (Scheme 3).

**Scheme 4:**
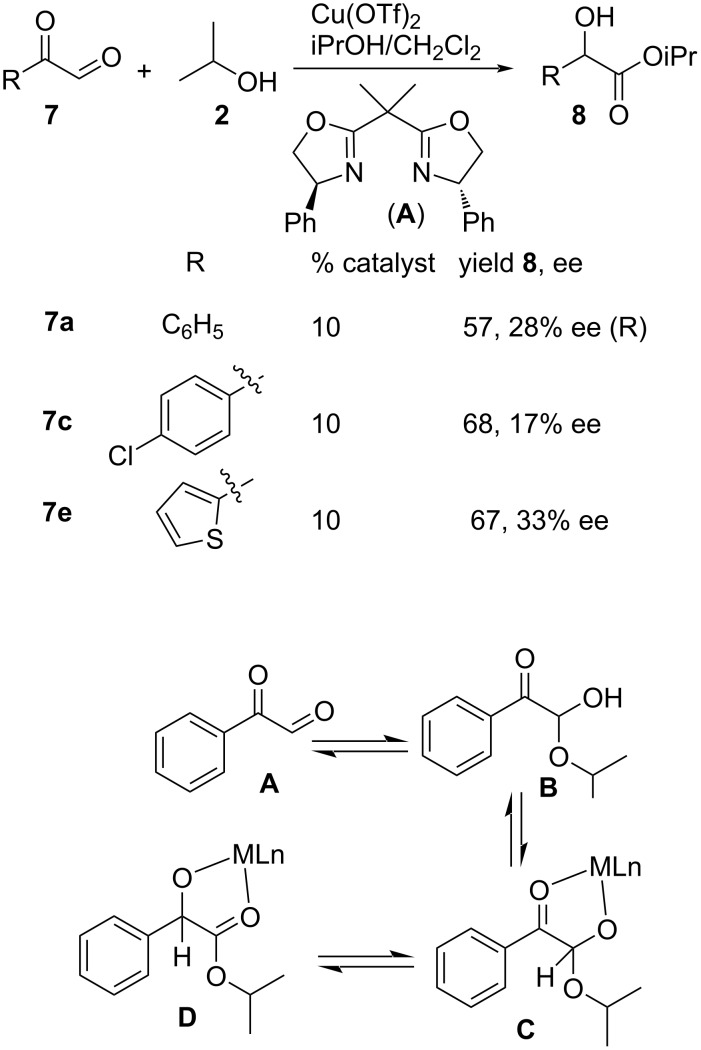
Cu(II)-PhBox-catalyzed asymmetric Cannizzaro reaction.

They also extended the approach to study enantioselective Cannizzaro reactions of similar substrates using a Cu bisoxazoline (**A**) [Cu(OTf)_2_-PhBox] complex as the chiral catalyst, producing the desired enantiomeric compounds in modest yields and up to 33% ee (Scheme 4). The mechanistic transformation of the aryl glyoxals is outlined below (Scheme 4), which depicts the coordination of the hemiacetal **B** with the metal catalyst to give **C**, followed by hydride transfer to form the metal-coordinated Cannizzaro product **D**.

**Scheme 5:**
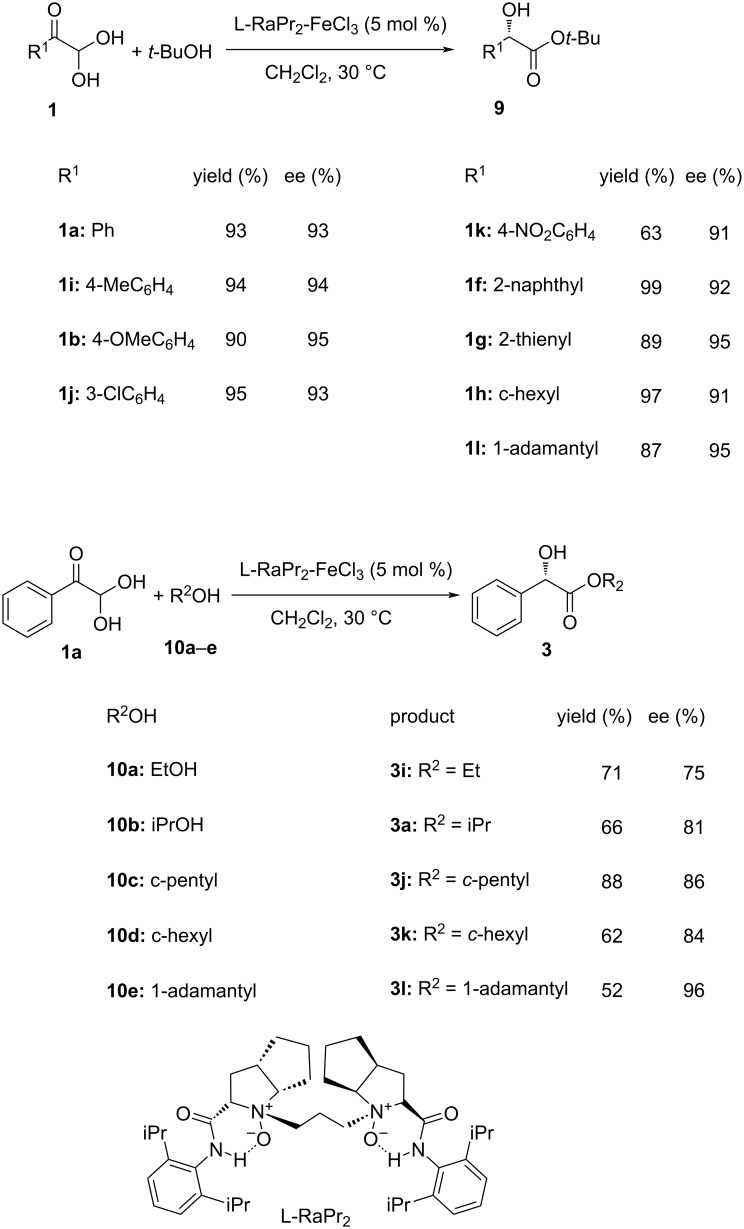
FeCl_3_-based chiral catalyst applied for the enantioselective intramolecular Cannizzaro reaction reported by Wu et al.

Another intramolecular asymmetric Cannizzaro reaction was reported by Wu et al. where they applied a FeCl_3_-based chiral catalyst with an *N,N′*-dioxide ligand [74]. The optimization of the reaction conditions revealed the L–RaPr_2_–FeCl_3_ complex being superior and delivering good to excellent results, thus witnessing a broad substrate scope taking different glyoxal monohydrates **1** and alcohols **10**. Excellent yields and enantioselectivities of the intramolecular Cannizzaro version were observed furnishing a wide range of alkyl and aryl mandelate esters **9** and **3** (Scheme 5).

**Scheme 6:**
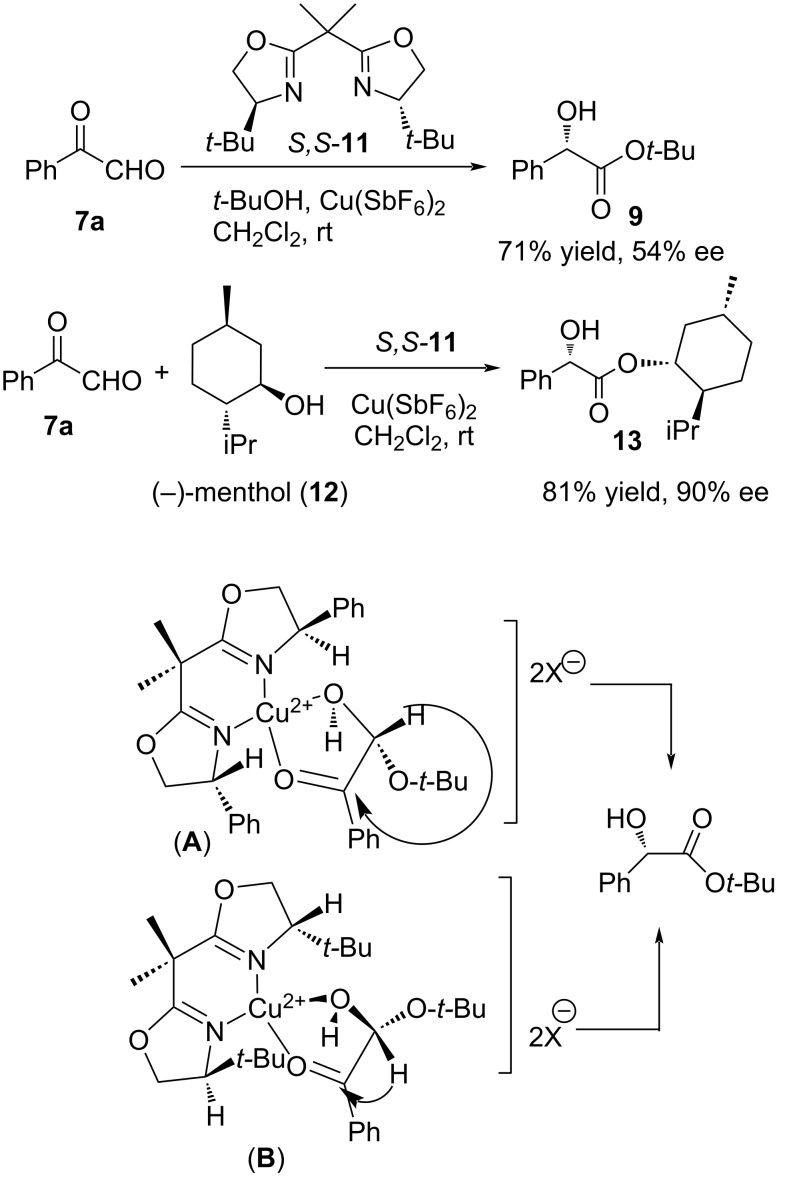
Copper bis-oxazoline-catalysed intramolecular Cannizzaro reaction and proposed mechanism.

The asymmetric intramolecular Cannizzaro reaction of anhydrous phenylglyoxal (**7a**) with alcohols was envisaged by Ishihara et al. using chiral copper bis(oxazoline) (*S,S*-**11**) catalysts to provide optically active mandelic acid esters **9** [75]. Among the different catalysts employed, Cu(SbF_6_)_2_ in the presence of *t*-butanol and (*S,S*)-bis oxazoline (*S,S***-11**), afforded the desired product in 71% yield and 54% enantioselectivity. They employed a double asymmetric induction with (+)/(−)-menthol (**12**), and CuX_2_ bis(oxazoline) catalyst where the corresponding chiral mandelate ester **13** was obtained in 81% yield and high selectivity (90% de) (Scheme 6). The proposed mechanism of the reaction is depicted below.

**Scheme 7:**
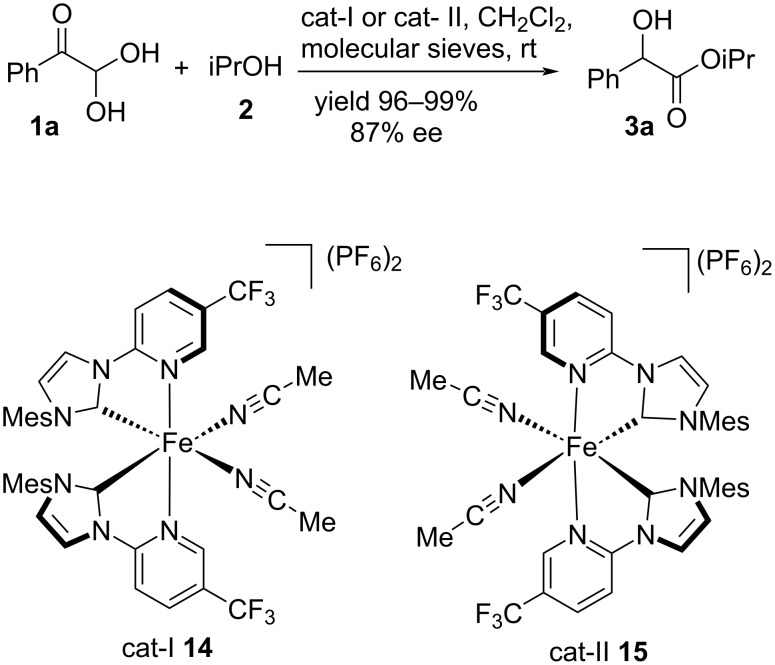
Chiral Fe catalysts-mediated enantioselective Cannizzaro reaction.

Hong et al. developed an asymmetric iron catalyst with the aim of expanding the platform of metal catalysis. Catalysts **14** and **15** proved to be effective in the transformation of glyoxal monohydrates **1a** and alcohol **2**, to deliver mandelate esters **3a** in good yields and enantioselectivities via an enantioselective intramolecular Cannizzaro reaction (Scheme 7) [76].

#### Lewis acid-catalyzed intermolecular Cannizzaro reactions

**Scheme 8:**
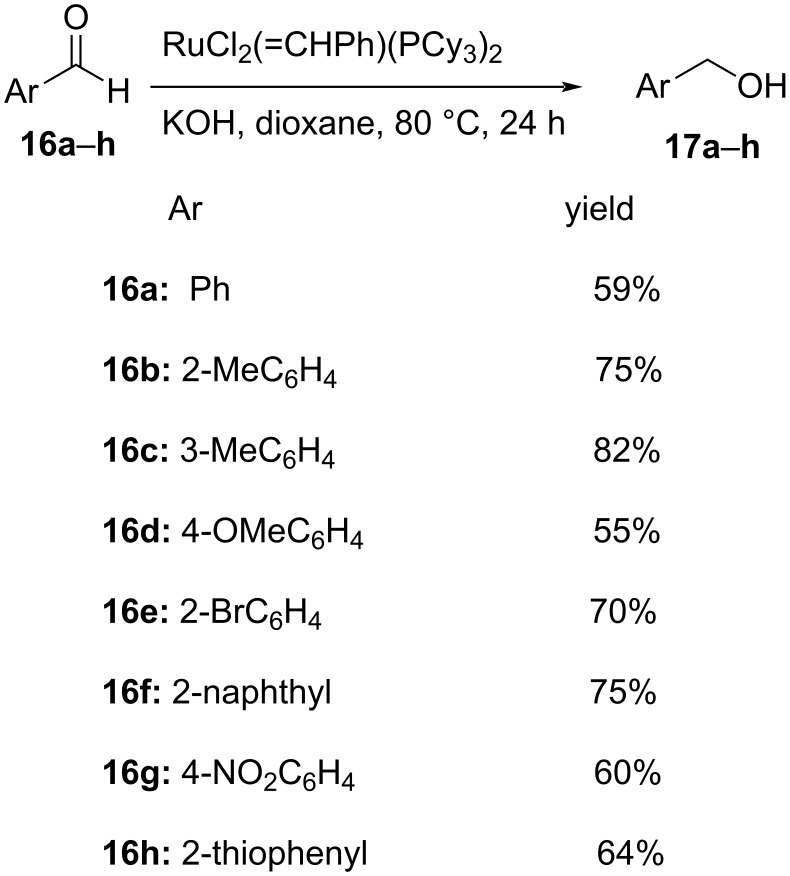
Ruthenium-catalyzed Cannizzaro reaction of aromatic aldehydes.

Kim et al. succeeded in the transformation of aromatic aldehydes **16** to the corresponding alcohols **17** using ruthenium catalysis in the presence of KOH and dioxane as solvent (Scheme 8). The reaction proceeded with modest to good yields, in the range of 40–82%, and depicts the Cannizzaro reaction in the transfer hydrogenation process [35].

**Scheme 9:**
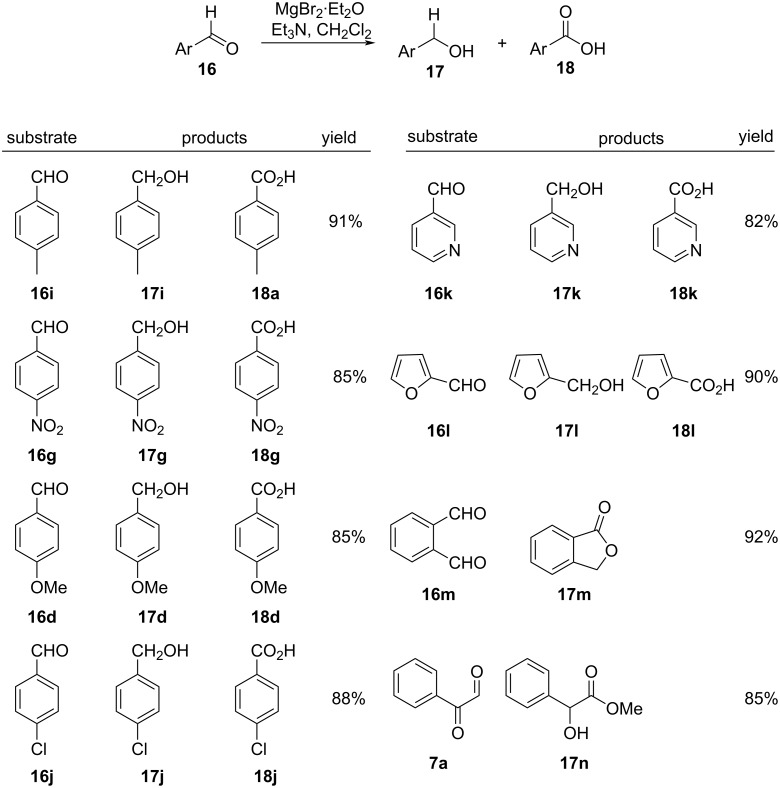
MgBr_2_·Et_2_O-assisted Cannizzaro reaction of aldehydes.

A facile room temperature Cannizzaro reaction protocol was established by Abaee et al. employing magnesium bromide etherate and triethylamine in dichloromethane [77]. The methodology afforded smooth transformation of aromatic aldehydes **16** to the corresponding alcohols **17** and carboxylic acids **18** in good yields (>80%). They also extended the methodology to dialdehydes such as phenylglyoxal (**7a**) and phthalaldehyde (**16m**), achieving an intramolecular version of the reaction to afford α-hydroxy acids and derivatives thereof (Scheme 9).

**Scheme 10:**
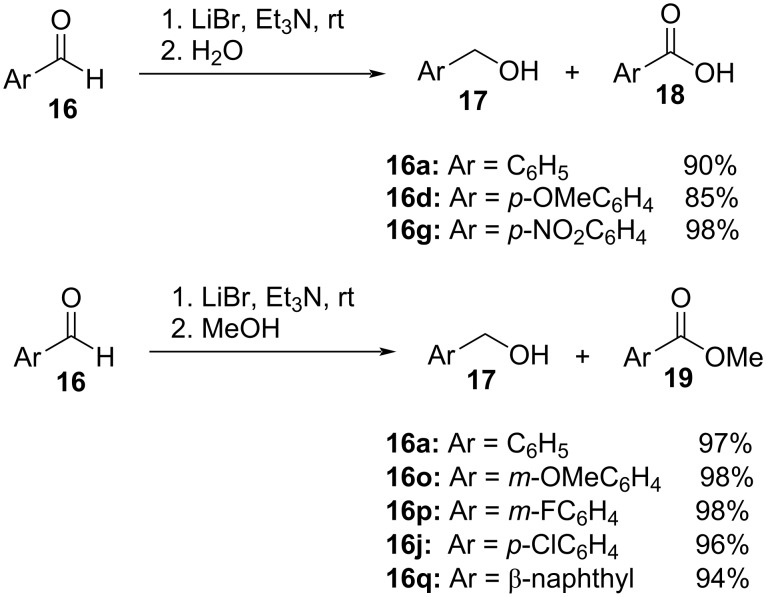
LiBr-catalyzed intermolecular Cannizzaro reaction of aldehydes.

They also devised a similar intermolecular Cannizzaro protocol using LiBr as the catalyst to achieve an analogous disproportionation of aromatic aldehydes **16** to the respective alcohols **17** and acids (**18**)/esters (**19**). The reaction proceeded with more than 85% yield in all cases with clean conversion to the products (Scheme 10) [78].

**Scheme 11:**
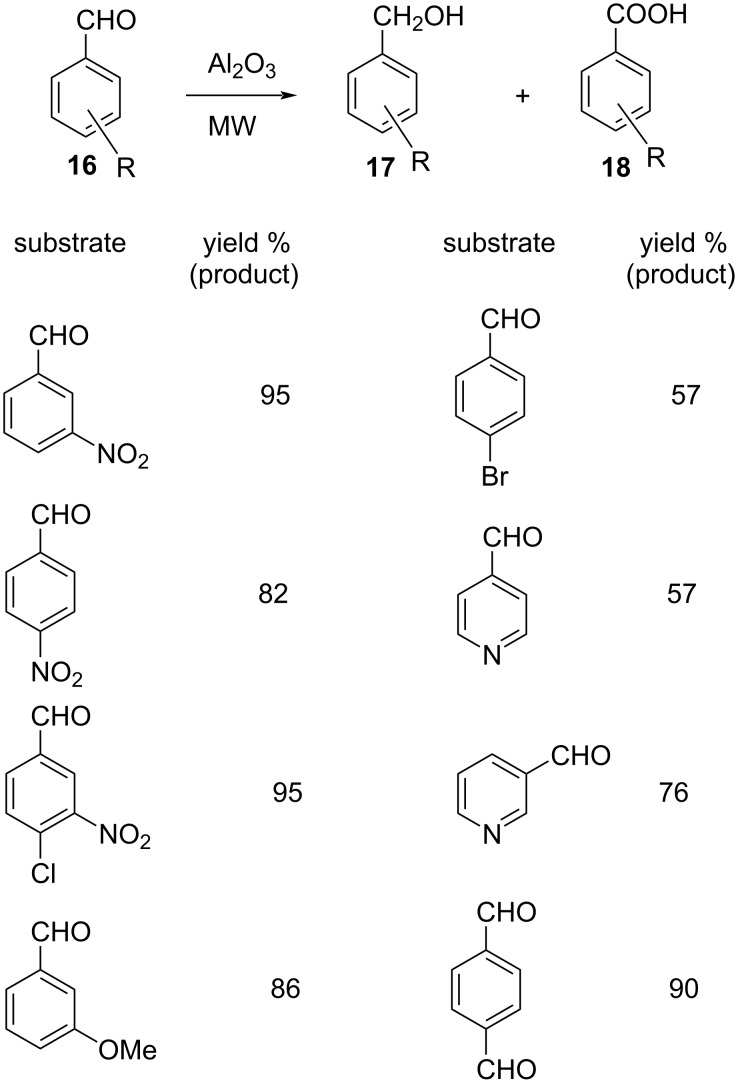
γ-Alumina as a catalyst in the Cannizzaro reaction.

The utility of neutral γ-alumina has been exploited as a polymeric Lewis acid catalyst in the Cannizzaro reaction of similar aromatic aldehydes **16**. The conversion to the respective aromatic alcohols **17** and carboxylic acids **18** was efficient as reflected in the modest to very good yields in each case (Scheme 11). The reaction proceeded under microwave conditions without the use of any base affording the desired Cannizzaro products [79].

**Scheme 12:**
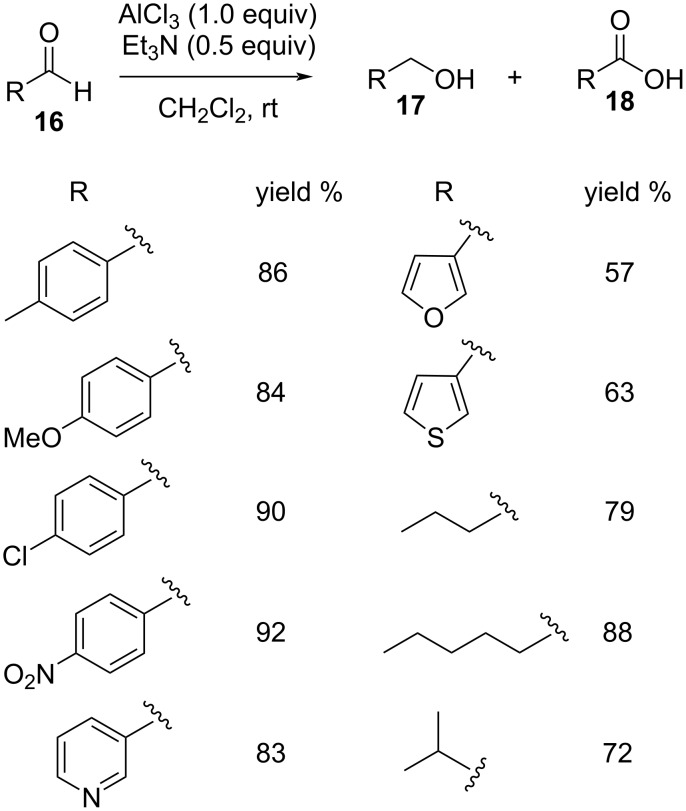
AlCl_3_-mediated Cannizzaro disproportionation of aldehydes.

An analogous Cannizzaro disproportionation was achieved by Sharifi et al. [80] where they made use of a catalyst system comprising AlCl_3_/Et_3_N in dichloromethane, transforming different aliphatic and aromatic aldehydes **16** to their end products. A wide variety of substrates were tested, using stoichiometric amounts of AlCl_3_ and transformed in good to excellent yields to the corresponding alcohols **17** and acids **18** (Scheme 12).

**Scheme 13:**
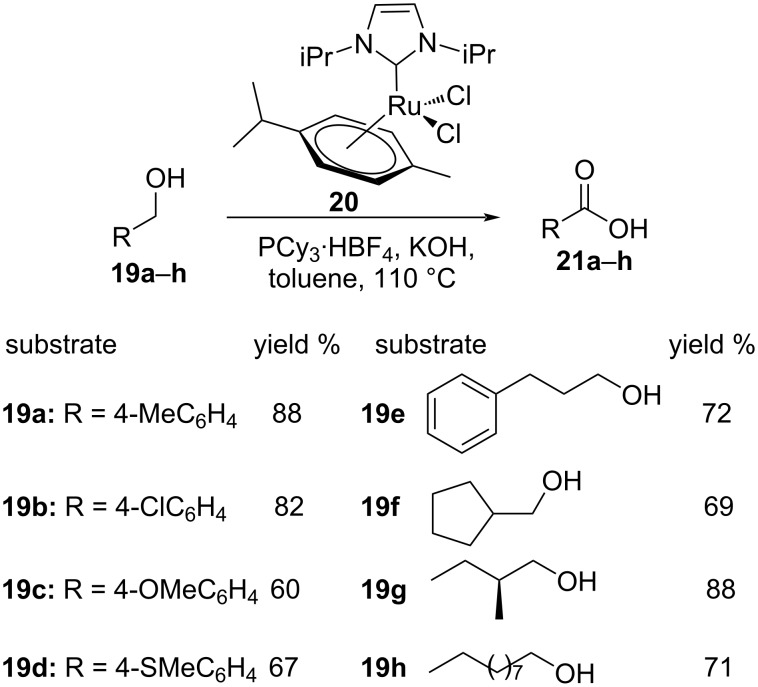
Ru–N-heterocyclic carbene catalyzed dehydrogenative synthesis of carboxylic acids.

**Figure 4:**
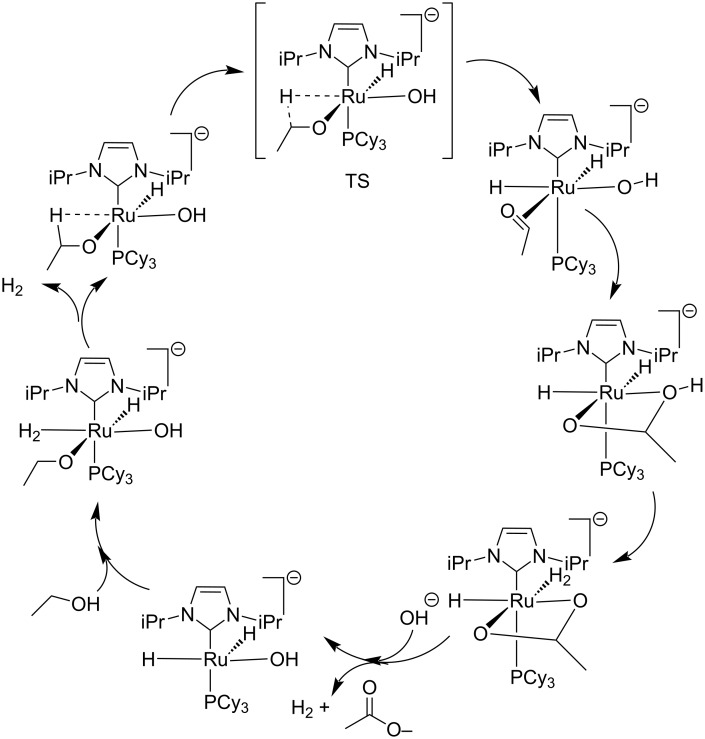
Proposed catalytic cycle for the dehydrogenation of alcohols.

Santilli et al. demonstrated a dehydrogenative protocol for the synthesis of carboxylic acids **21** from primary alcohols **19** employing a ruthenium *p*-cymene complex (**20**) in the presence of a suitable hydroxide [81]. Both primary aliphatic alcohols and benzylic alcohols delivered fruitful results. However, the reaction of benzyl alcohols was found to proceed within shorter reaction times and much higher yields compared to aliphatic alcohols. This process presumably involves a Cannizzaro reaction during the conversion of the benzyl alcohols and the intermediate aldehyde reacts with the hydroxide under the Cannizzaro conditions to give the desired product (Scheme 13). The mechanistic pathway for the transformation is represented in Figure 4.

#### Desymmetrization via intramolecular Cannizzaro reaction

**Scheme 14:**
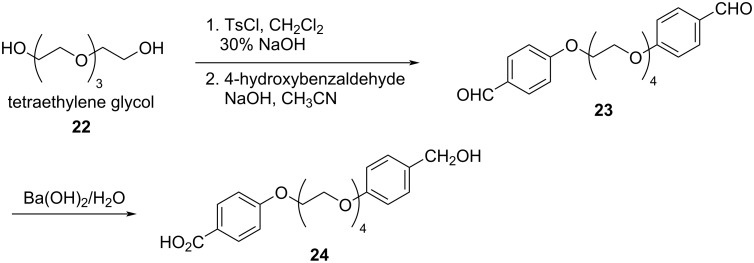
Intramolecular desymmetrization of tetraethylene glycol.

Vida et al. reported the intramolecular Cannizzaro reaction of dialdehyde **23**, synthesized from tetraethylene glycol (TEG) **22**. The dialdehyde **23** underwent a clean desymmetrization to form the hydroxy carboxylic acid derivative **24**. The reaction was mediated by Ba^2+^ which perfectly bound the TEG and allowed the aldehyde functionalities to be placed in the appropriate vicinity for the reaction to take place in appreciably good yields (84%). The barium cation template is the key for the reaction, as is the base concentration for effective hydride transfer (Scheme 14) [29].

**Scheme 15:**
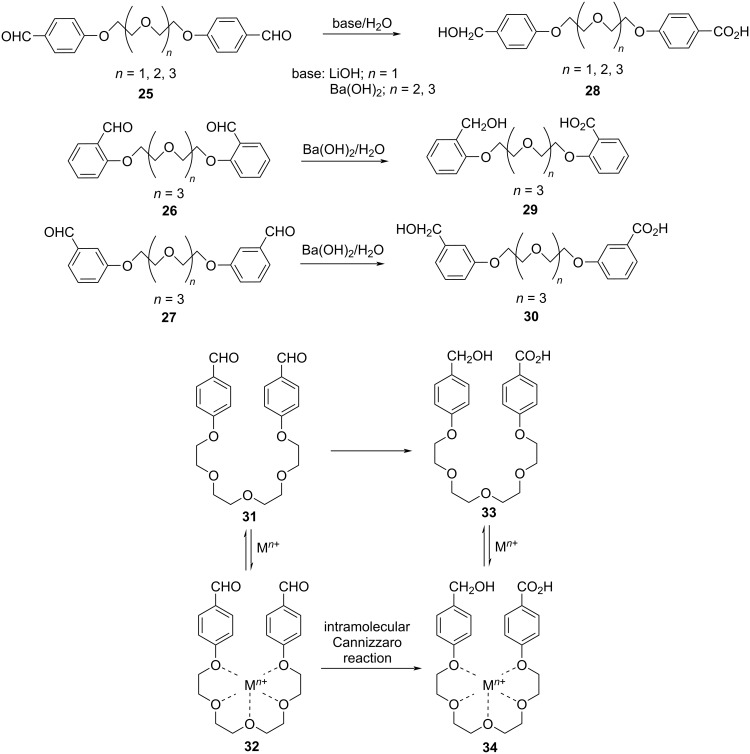
Desymmetrization of oligoethylene glycol dialdehydes.

They also extended the scope of the intramolecular Cannizzaro reaction to ethylene glycol units of different chain lengths ranging from 2–5 (**25**, **26**, and **27**), by varying the aromatic substitution in the ortho, meta and para-positions and finally obtained the desymmetrized products **28**, **29**, and **30** in good to excellent yields (Scheme 15). This has been effectively depicted in the proposed strategy where the metal ion acts as the binding cation template for the intramolecular desymmetrization (Scheme 15) [82].

**Scheme 16:**
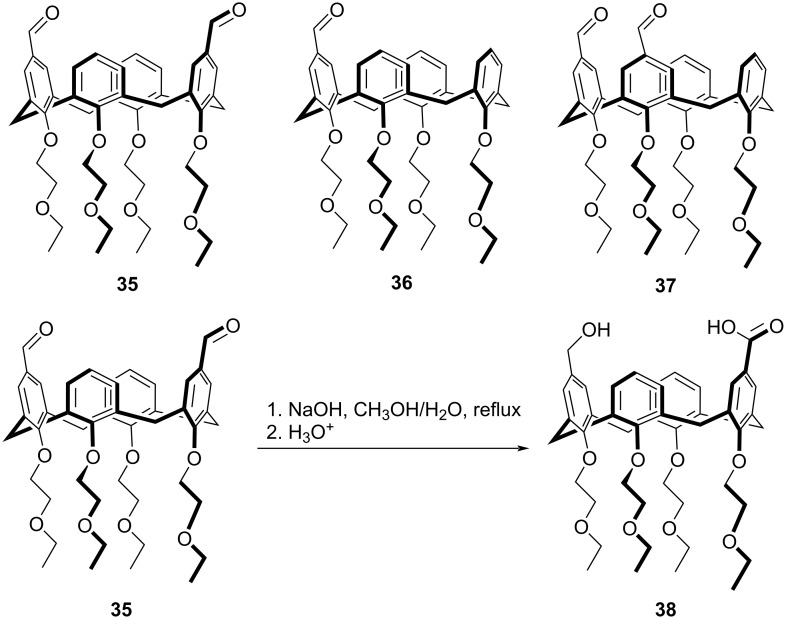
Intramolecular Cannizzaro reaction of calix[4]arene dialdehydes.

A similar highly efficient intramolecular Cannizzaro reaction of calix[4]arene dialdehydes was observed by Galli et al. where the 1,3-distal cone **35** significantly responded to Cannizzaro disproportionation, forming the hydroxy acid product **38**, using a strong base [83]. On the other hand, the analogous 1,2-vicinal isomer **36** and the monoaldehyde **37** failed to produce any fruitful results. This difference in reactivity was referred to the relative positions of the formyl groups in the respective isomers where the geometry of the 1,3-distal dialdehyde **35** was conformationally favorable for the intramolecular hydride attack to take place leading to the formation of the product (Scheme 16).

**Scheme 17:**
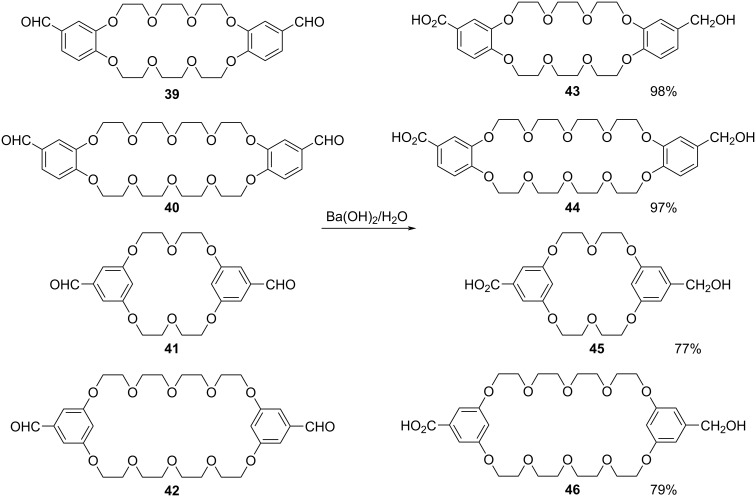
Desymmetrization of dialdehydes of symmetrical crown ethers using Ba(OH)_2._

Symmetrical crown ethers having two aldehyde groups **39**–**42** were functionalized and desymmetrized by Rouser et al. using an intramolecular Cannizzaro reaction to give the corresponding unsymmetrical acid–alcohol substituted crown ether derivatives **43–46**. Good to excellent yields of the desymmetrized Cannizarro products **43**–**46** were obtained using Ba(OH)_2_ as the base, thus effecting efficient desymmetrization of crown ether dialdehydes (Scheme 17) [84].

#### Synthesis of natural products and pharmaceuticals

**Exploring intramolecular Cannizzaro reaction:** The Cannizzaro reaction is a versatile synthetic tool with applications in the synthesis of natural products, fine chemicals, and pharmaceuticals. Its ability to introduce carboxylic acid functionalities and facilitate functional group interconversions makes it a valuable method for chemists engaged in the design and synthesis of diverse organic compounds. A selection of applications is depicted herein.

**Scheme 18:**
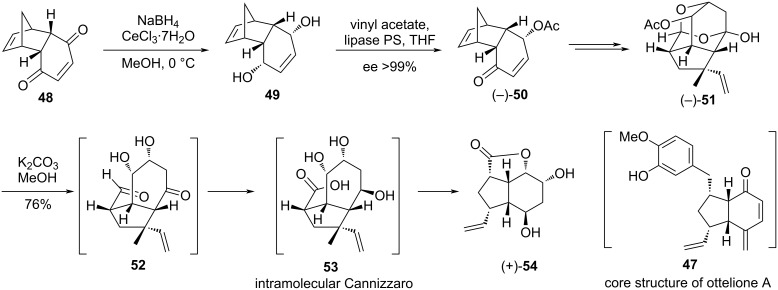
Synthesis of ottelione A (proposed) via intramolecular Cannizzaro reaction.

Mehta et al. established a highly selective intramolecular Cannizzaro reaction while accomplishing the synthesis of the bicyclic core structure of proposed ottelione A (**47)** [85]. Commencing from the Diels–Alder adduct **48**, an enzymatic desymmetrization of the reduced diol **49** formed the enantiopure **50** (ee >99%). A cascade of reaction sequences delivered the tetracyclic cage compound **51**. Acetal opening in **51** afforded the keto-aldehyde **52** which underwent an intramolecular Cannizzaro reaction to give the trihydroxy acid **53**, finally cyclizing to the lactone diol **54**, elaboration of which led to the desired target (Scheme 18).

**Scheme 19:**
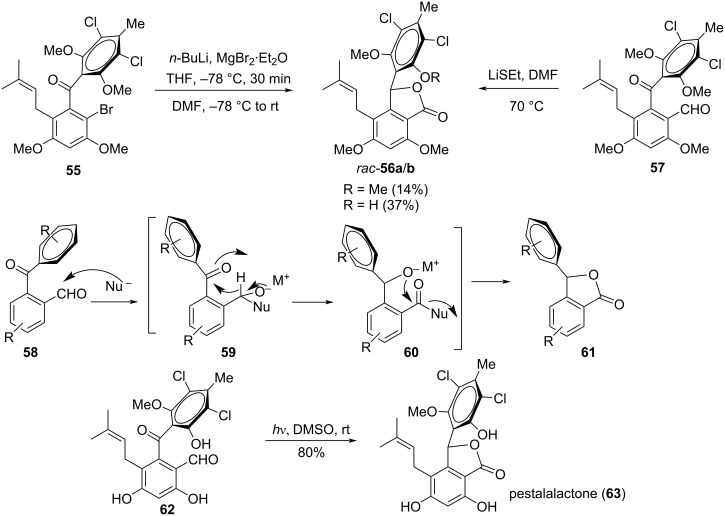
Intramolecular Cannizzaro reaction for the synthesis of pestalalactone.

An interesting application of the intramolecular Cannizzaro reaction was demonstrated by the group of Schmalz in the total synthesis of the marine antibiotic pestalone [86]. They observed a facile isomerization of the pestalone derivatives **55/57** into the intramolecular lactone derivatives rac-**56a**,**b** which features a Cannizzaro–Tishchenko-type reaction representing analogous derivatives related to pestalachloride A. The mechanistic transformation could be illustrated schematically following the transformation from **58** to **61** through the intermediates **59** and **60**. The natural pestalone **62** when subjected to a photo-induced transformation leads to pestalalactone (rac-**63**), involving a photo-induced Cannizzaro–Tishchenko sequence (Scheme 19).

**Scheme 20:**
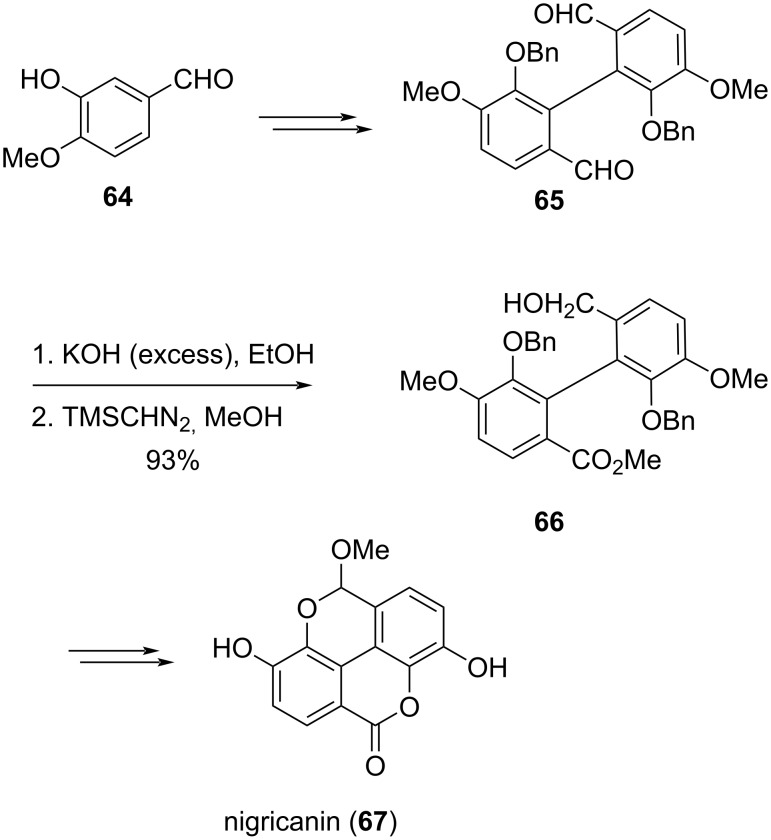
Synthetic strategy towards nigricanin involving an intramolecular Cannizzaro reaction.

An efficient synthetic strategy for nigricanin was accomplished by Abe et al. wherein they utilized the intramolecular Cannizzaro reaction as a key step for desymmetrization of the crucial dialdehyde intermediate **65** [87]. The sequence of transformations commencing from the aldehyde **64** afforded the desymmetrized biaryl derivative **66** and proceeded towards the final natural product **67** (Scheme 20).

**Scheme 21:**
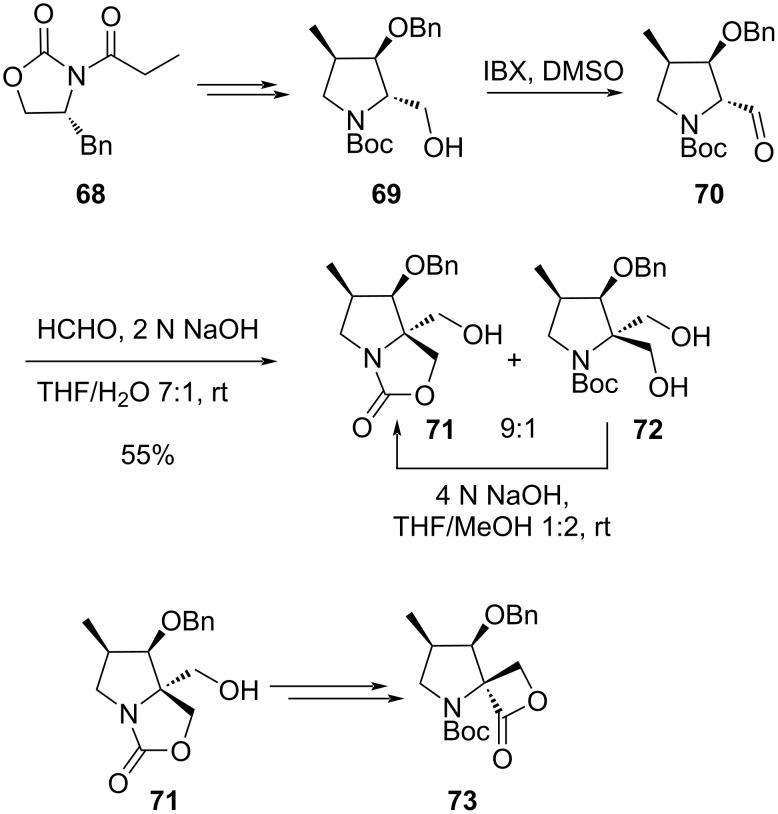
Spiro-β-lactone-γ-lactam part of oxazolomycins via aldol crossed-Cannizzaro reaction.

**Applying crossed-Cannizzaro reaction:** Mondal and coauthors demonstrated an efficient application of the aldol/crossed-Cannizzaro reaction in the construction of the spiro-β-lactone ring while targeting the spiro-β-lactone-γ-lactam ring of oxazolomycin and lazollamycin [88]. Proceeding towards the requisite fragment they envisaged a series of crucial diastereoselective transformations arriving at the precursor **69** to the Cannizzaro reaction commencing from **68**. The primary hydroxymethyl functionality in **69** was oxidized to the corresponding aldehyde **70**, which was subsequently treated with 37% aqueous formaldehyde and NaOH, to result in a mixture of the *gem*-hydroxymethyl derivative **72** and the carbamate **71** which led to the spiro-β-lactone core **73** (Scheme 21).

**Scheme 22:**
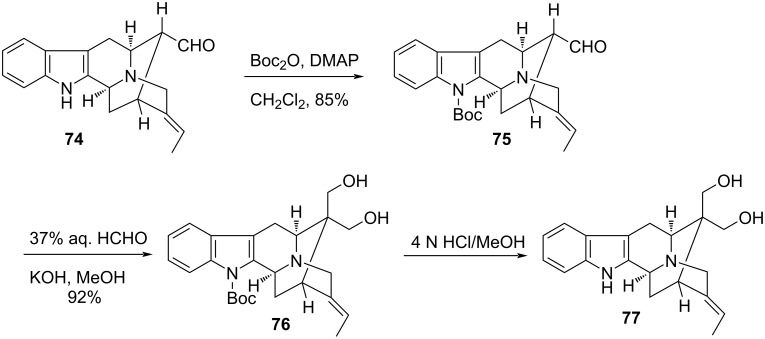
Synthesis of indole alkaloids via aldol crossed-Cannizzaro reaction.

An expedient use of the Cannizzaro reaction was exemplified in the noteworthy enantioselective synthesis towards the indole alkaloids 16-epivellosimine, (+)-polyneuridine, and (+)-macusine A as reported by Cook and coworkers [89]. This was effectively worked out from ᴅ-(−)-tryptophan via the common intermediate, (+)-vellosimine (**74**). The protocol reflected the stereocontrolled formation of the C-16 quaternary center in **76** created via an intermolecular crossed-Cannizzaro reaction of **75**, generated from **74**, using 37% aqueous formaldehyde. The quaternization proceeded in excellent yield (92%) and formed the diol **77**, after the removal of the Boc-protecting group, where the prochiral hydroxymethyl groups ultimately paved the way towards the natural products (Scheme 22).

**Scheme 23:**
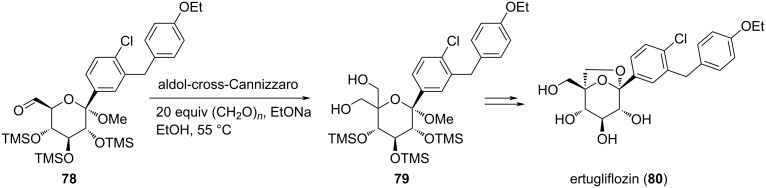
Aldol and crossed-Cannizzaro reaction towards the synthesis of ertuliflozin.

Bernhardson and coworkers developed a simple scalable route towards ertugliflozin (**80**), a *C*-glycoside containing a bicyclic ketal motif. The method illustrates the potent use of an aldol-crossed-Cannizzaro reaction to form the quaternarized pentol **79** from the aldehyde **78** in 94% yield and >99.8% purity after recrystallization. This symbolizes the efficient applicability of the Cannizzaro reaction for the synthesis of medicinally useful molecules (Scheme 23) [90].

The Cannizzaro reaction has also been applied to the preparation of mandelic acid-based synthons, which gain potential importance in the synthesis of prostaglandins, cephalosporins, and homatropine [65–69].

### Synthesis of useful scaffolds

**Scheme 24:**
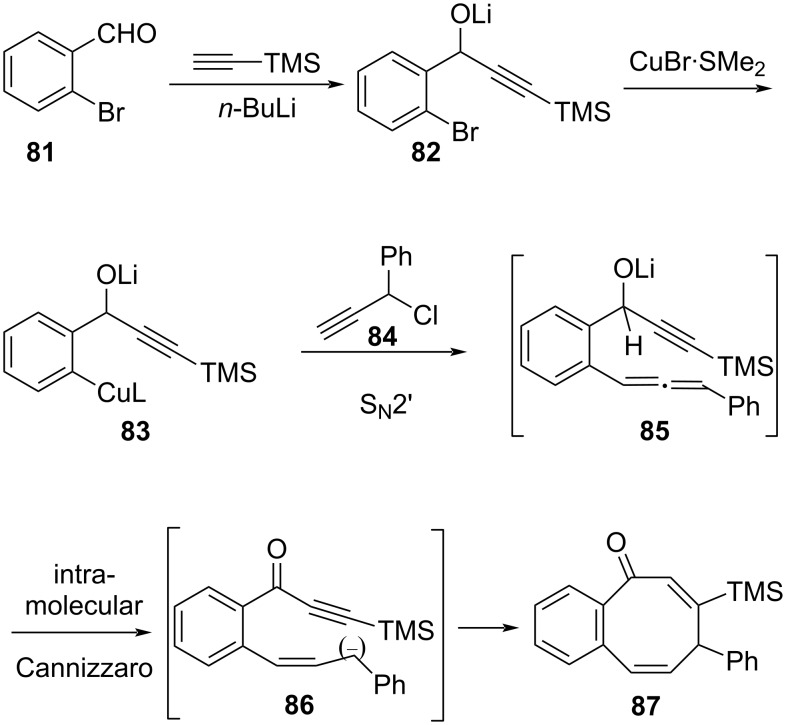
Synthesis of cyclooctadieneones using a Cannizzaro reaction.

Burroughs et al. developed an intramolecular Cannizzaro-based cascade synthesis for the construction of 8-membered cycloocta-2,5-dienones [91]. The initial formation of the organolithium species **82** formed by acetylide addition to the ortho-substituted bromoaldehyde **81**, was subjected to halogen exchange and transmetalation to the organocuprate **83**. The latter undergoes an S_N_2’ addition to the propargyl chloride **84** and the resulting allene intermediate **85** undergoes an intramolecular Cannizzaro-type hydride transfer via **86** to produce the 8-membered cyclized target **87** in good yield (70%) (Scheme 24).

**Scheme 25:**
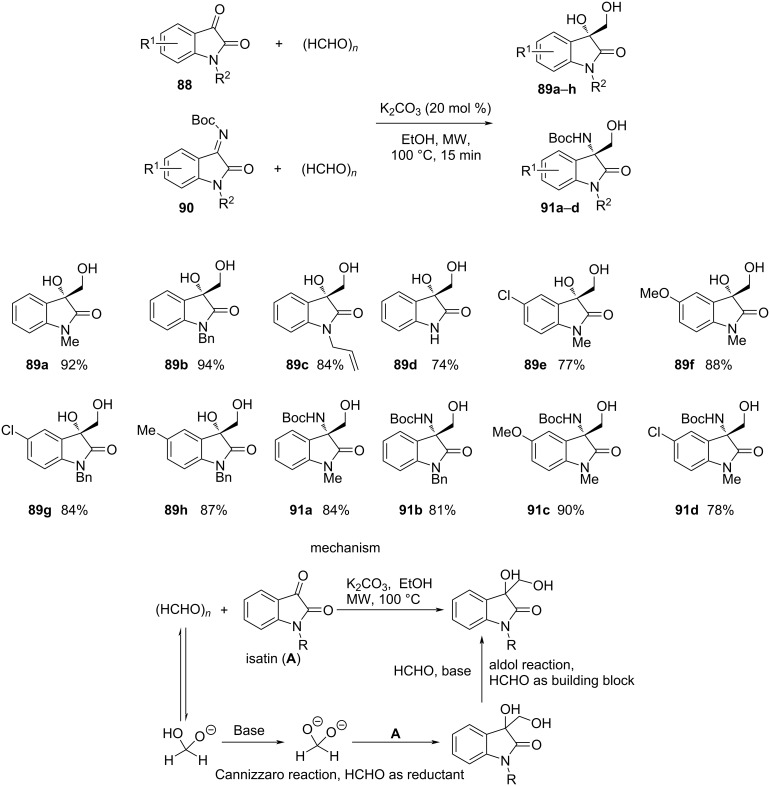
Microwave-assisted crossed-Cannizzaro reaction for the synthesis of 3,3-disubstituted oxindoles.

Huang et al. set up a clean crossed-Cannizzaro-aldol reaction strategy of isatin derivatives **88** and **90** with paraformaldehyde under microwave irradiation to furnish 3,3-disubstituted oxindole derivatives **89a**–**h** and **91a**–**d** [92]. The representative 3-hydroxymethyloxindole adducts with varying substituents (R^1^ and R^2^) were obtained in good to excellent yields witnessing the feasibility of the methodology (Scheme 25). The mechanism depicting the proposed strategy for the Cannizzaro-aldol transformation involves an initial Cannizzaro reaction between paraformaldehyde and the isatin substrate, followed by an aldol transformation to the final product.

**Scheme 26:**
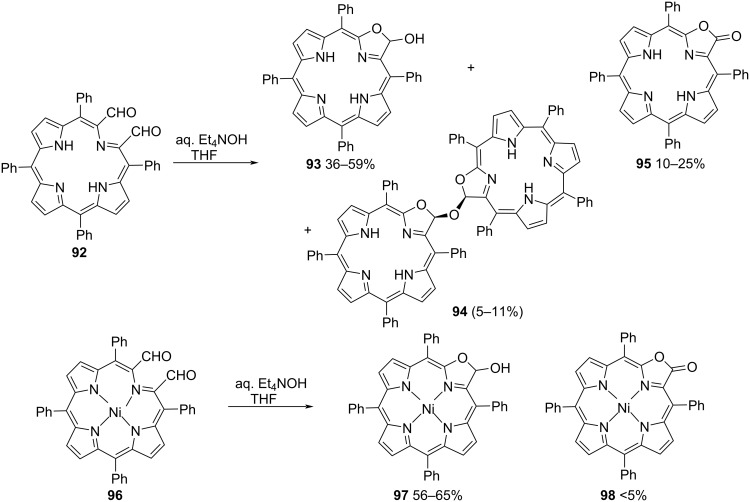
Synthesis of porphyrin-based rings using the Cannizzaro reaction.

Bruckner and coworkers synthesized pyrrole-modified porphyrin ring systems from secochlorin bisaldehydes, representing an interesting application of the intramolecular Cannizzaro reaction. Subjecting the bisaldehyde **92** to basic conditions using Et_4_NOH, formed the product **93** in 36–59% yield along with the overoxidized compound **95**, and the dimer **94** as byproducts. However, upon reaction with methanol under acidic conditions, the product **93** and the dimer **94** underwent a smooth transformation to the methyl acetal in high yields. Similar exposure of the nickel(II) complex **96** to the same basic conditions resulted in the formation of the Cannizzaro adduct hemiacetal Ni(II) complex **97** in 56–65% yield predominantly with the byproduct **98** in less than 5% yield (Scheme 26) [93].

**Scheme 27:**
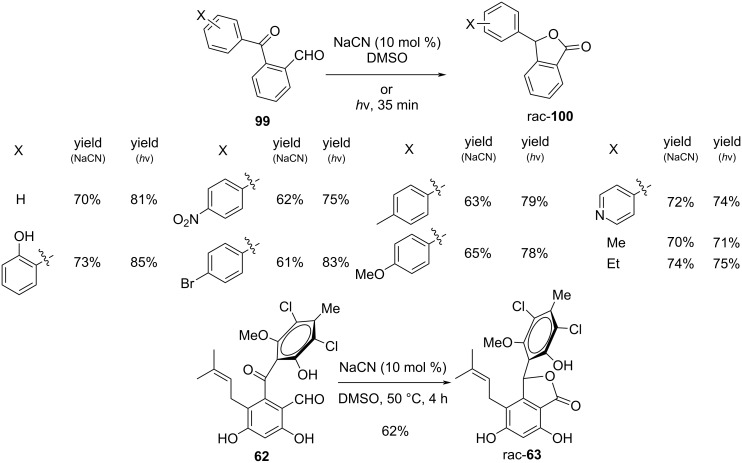
Synthesis of phthalides and pestalalactone via Cannizarro–Tishchenko-type reaction.

Schmalz and coworkers transformed 2-formylarylketones **99** into 3-substituted phthalides **100**, as evidenced by the per-*O*-methylated derivative of pestalone, a marine natural substance. Either in a Cannizarro–Tishchenko-type reaction with nucleophile catalysis (NaCN) or under photochemical conditions (DMSO, 350 nm), the transformation often proceeds without any problems in DMSO (Scheme 27) [94].

**Scheme 28:**
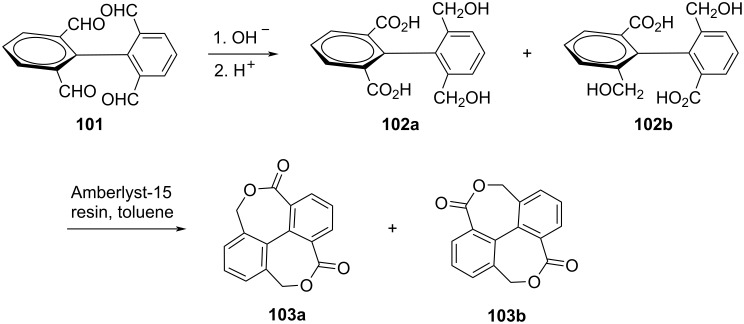
Synthesis of dibenzoheptalene bislactones via a double intramolecular Cannizzaro reaction.

Two isomeric bislactones 4,6,10,12-tetrahydro-4,12-dioxo-5,11-dioxadibenzo[*ef*,*kl*]heptalene (**103a**) and 4,6,10,12-tetrahydro-4,10-dioxo-5,11-dioxadibenzo [*ef*,*kl*]heptalene (**103b**) were synthesized [95] as chiral and atropisomeric molecules via double intramolecular Cannizzaro reaction of 1,1'-biphenyl-2,2',6,6'-tetracarboxaldehyde (**101**) (Scheme 28).

## Conclusion

The Cannizzaro reaction is one of the oldest reactions in organic chemistry for the synthesis of acid and alcohol functionalities through disproportionation reaction of non-enlizable aldehydes. Apart from the conventional methods, several modern modifications using mild and sustainable reagents, solvents, advanced instrumental techniques, and catalysts in both intramolecular and intermolecular versions of the reaction have made the Cannizzaro reaction an important tool for the synthesis of diverse molecules containing alcohol, acid, ester, and amide, etc. functionalities. The vast area of synthetic venture highlights the significance of this reaction, as exemplified here, in some of the most recent advances of this reaction during the last two decades. Proper utilization of Lewis acid catalysis, desymmetrization of symmetrically remote dialdehyde molecules, synthesis of bioactive natural products like oxazolomycin, ottelione A, pestalalactone, nigricanin and other useful molecules of potential interest such as oxindoles, cyclooctadienones, mandelic acid derivatives have been represented. The high yielding methodologies, emphasizing different greener perspectives, are evident in every case and reflect the inner potential of the Cannizzaro reaction in accomplishing the synthesis of a diverse series of molecular entities. Being the first of its kind, this review presents a comprehensive outlook of the Cannizzaro reaction in several aspects. The application of this highly valuable reaction to the functionalization of bioactive molecules with improved synthetic conditions, will broaden its use in the future.

## Data Availability

Data sharing is not applicable as no new data was generated or analyzed in this study.
